# Structural racism, air pollution and the association with adverse birth outcomes in the United States: the value of examining intergenerational associations

**DOI:** 10.3389/fepid.2023.1190407

**Published:** 2023-06-22

**Authors:** Miatta A. Buxton, Nancy L. Fleischer, Annie Ro, Marie S. O’Neill

**Affiliations:** ^1^ Department of Epidemiology, School of Public Health, University of Michigan, Ann Arbor, MI, United States; ^2^ Department of Health, Society, and Behavior, Program in Public Health, University of California, Irvine, Irvine, CA, United States; ^3^ Department of Environmental Health Sciences, School of Public Health, University of Michigan, Ann Arbor, MI, United States

**Keywords:** structural racism, air pollution, intergenerational associations, existing data sources, adverse birth outcomes

## Abstract

Structurally racist policies and practices of the past are likely to be a driving factor in current day differences in exposure to air pollution and may contribute to observed racial and ethnic disparities in adverse birth outcomes in the United States (U.S.). Non-Hispanic Black women in the U.S. experience poorer health outcomes during pregnancy and throughout the life course compared to non-Hispanic White women. This disparity holds even among non-Hispanic Black women with higher socioeconomic status. Reasons for this finding remain unclear, but long-term environmental exposure, either historical exposure or both historical and ongoing exposure, may contribute. Structural racism likely contributes to differences in social and environmental exposures by race in the U.S. context, and these differences can affect health and wellbeing across multiple generations. In this paper, we briefly review current knowledge and recommendations on the study of race and structural racism in environmental epidemiology, specifically focused on air pollution. We describe a conceptual framework and opportunities to use existing historical data from multiple sources to evaluate multi-generational influences of air pollution and structurally racist policies on birth and other relevant health outcomes. Increased analysis of this kind of data is critical for our understanding of structural racism's impact on multiple factors, including environmental exposures and adverse health outcomes, and identifying how past policies can have enduring legacies in shaping health and well-being in the present day. The intended purpose of this manuscript is to provide an overview of the widespread reach of structural racism, its potential association with health disparities and a comprehensive approach in environmental health research that may be required to study and address these problems in the U.S. The collaborative and methodological approaches we highlight have the potential to identify modifiable factors that can lead to effective interventions for health equity.

## Introduction

Structural racism may play a role in observed disparities in adverse birth outcomes by different racial groups in the United States (U.S.). Structural racism, described as “the larger system of policies, practices, ideologies, and institutions that foster racial inequality by creating differential access to resources and opportunities” (1), is reported to be a major driver of the uneven distribution of air pollution across different racial and ethnic minoritized groups in the U.S in both the short and long term (2–4). The impact of these disparities may be even greater when consideration is given to the historical context of structural racism and corresponding data are incorporated in study designs. A comprehensive approach to understanding factors that may contribute to disparities in adverse birth outcomes will be crucial for the development of effective intervention strategies in the U.S.

Race, or the division of the population by racially defined subgroups, has been described as a socially constructed undertaking that does not have a biological basis (5, 6) and may not be causally associated with disparities observed in key health outcomes such as adverse birth outcomes. Instead, policies and practices such as redlining, a government-backed practice in the 1930s that led to the classification of neighborhoods with a high percentage of Black individuals and immigrants as “hazardous” and consequently, considered a low investment priority by lenders (2), may be a key component driving observed differences in birth outcomes. Maps created by the Home Owners' Loan Corporation (HOLC), a government-sponsored entity intended to assist homeowners at risk of foreclosure, informed this practice. The HOLC utilized the following system to classify neighborhoods: Green = “Best”; Blue = “Still Desirable”; Yellow = “Declining”; Red = “Hazardous” (7). This practice led to disinvestment in neighborhoods that were redlined or classified as “red”, which subsequently led to major declines in neighborhood quality and lost opportunities for home ownership and wealth accumulation (8). With neighborhood decline, land value may have been affected and this may have created conditions that were favorable to industries that produce pollution (9). In addition, surrounding areas near redlined neighborhoods were zoned as “industrial”, thereby allowing for the siting of major pollution producing sources (10). Despite officially ending in 1968 (11), redlining's impact has persisted and links exist between redlined neighborhoods and the siting of present-day pollution sources such as roadways, industrial facilities and power plants (2, 12). Additionally, environmental justice scholars and activists in the United States have documented that exposure to air pollution and other adverse environmental conditions are often disproportionately higher among racially minoritized groups (13), despite lower consumption of goods and services associated with the production of air pollution (14). In addition, associations have been examined between redlining and current day air pollution levels in cities across the U.S (2). Although air pollution concentrations have declined over time, disparities have persisted (4). Exposure remains a problem from an environmental justice point of view because even levels below health-based regulatory standards are associated with adverse health outcomes (15, 16).

Despite increasing evidence that redlining and other important historical policies are associated with disparities in health, educational and economic outcomes, particularly given the pervasive and persistent nature of structural racism, very few studies focus on intergenerational associations (17). In environmental epidemiology, the lack of widespread use of methodological approaches that incorporate intergenerational exposures and measures of structural racism may partly be due to logistical complexity in finding and linking datasets across generations. The first goal of this article is to provide context about studying racial disparities, structural racism and environmental epidemiology. Then, we review evidence on air pollution associations with adverse birth outcomes. In addition, we discuss a conceptual framework that informs the rationale behind our recommendation to evaluate associations between intergenerational exposure to air pollution and adverse birth outcomes. We also identify examples of existing historical data from multiple sources to evaluate these intergenerational associations and to identify factors, including structurally racist policies that differ across communities, which may provide opportunities for intervention. In this manuscript, we focus on non-Hispanic Black and non-Hispanic White persons because the largest disparities in adverse birth outcomes such as preterm birth, low birth weight and infant mortality are found among non-Hispanic Black individuals compared to non-Hispanic White individuals. However, the methods here would be applicable and equally important in studying health disparities affecting other racially minoritized groups. Ultimately, the intended purpose of this manuscript is to provide an overview of the current state of structural racism in environmental epidemiology, introduce a conceptual framework of intergenerational exposures, and offer data considerations when taking an intergenerational approach in environmental epidemiological research.

## Studying race and structural racism and current practice in environmental epidemiology

Many studies evaluating differences in exposure to environmental pollution or adverse birth outcomes compare a racially minoritized group or groups to non-Hispanic White persons as the reference group (18). Although useful for highlighting differences by racially minoritized groups with non-minoritized groups, continued comparisons of health outcomes by race without a discussion of the myriad of factors that race represents may lead to incorrect conclusions or perpetuation of the idea that observed disparities may be due to biological differences and thus limit potential opportunities for intervention. Instead, health disparities by racially classified categories are likely in many cases to result from longstanding exclusionary and racist systems and practices (19). Therefore, using rigorous study designs that illuminate the impact of these systems will ultimately inform policies needed to change these systems and will likely advance the field in meaningful ways (20). The use of multilevel and multidimensional models (e.g., use of factor analysis to combine multiple dimensions of structural racism) (21); mixed data (e.g., individual level biological data and administrative data, or mixing of quantitative and qualitative data, interactions among structural and personal levels of racism) (17); and life course and longitudinal analyses, have been recommended as a way to move health disparities research forward (19).

Specific to environmental epidemiology, Hicken and colleagues recently reviewed literature on race variables as an effect modifier of the air pollution and mortality association in the U.S. as a way of illustrating current practice and areas for improvement on the conceptualization of race in the environmental health literature (22). They found a pattern in which the reviewed studies provided little discussion conceptualizing and justifying the use of race in the study, and few discussions of reasons behind any observed effect modification by race. Indeed, the authors suggest that frameworks that include social meaning of race and positioning of race within structural and cultural systems are lacking in the air pollution and mortality literature, and call for greater “rigor and intention” in the way race is considered in such studies. One example they provide is looking at the role of “place within race categories” as a way of identifying features that differ by place and their role in exposure/outcome differences. They also call for framing of research questions in a way that is grounded in the humanities literature and has clear policy and intervention implications. Similar calls were given in a recent editorial emphasizing interest in publishing research on environmental racism in environmental health sciences research and introducing new guidelines to enhance the rigor of such research (23).

## Air pollution and adverse birth outcomes—Conceptual framework for intergenerational studies

Several individual epidemiologic studies and meta-analyses support associations between higher levels of air pollution and adverse birth outcomes (24–26). Associations are hypothesized to occur via multiple mechanisms, including oxidative stress, inflammation, and endothelial dysfunction (27). Air pollution is a risk factor for adverse birth outcomes, such as low birth weight (26) and preterm birth (25, 28, 29). Such birth outcomes occur at higher rates among non-Hispanic Black compared to non-Hispanic White women (30), and persist even after accounting for important factors such as education and socioeconomic status (31, 32). Reasons for this finding remain unclear, but long-term air pollution exposure, either historical or both historical and ongoing exposure to air pollution, may partly contribute. It is thus crucial for the underlying factors, including systemic factors, which affect exposure to air pollution to be investigated in any serious attempts to address health disparities in the U.S. Previous and ongoing stressors may operate via the “weathering hypothesis”, which posits that combined and repeated exposure to social and environmental stressors over the life course may lead to accelerated declines in health (33) and may help explain the mechanisms involved in the cumulative potential of harmful exposures. Although weathering can affect anyone who is continually exposed to stressors, including non-Hispanic White individuals, the effects are more pronounced among non-Hispanic Black individuals (34, 35). An important consideration for understanding the broader systems and processes that may contribute to persistent disparities in adverse health outcomes between non-Hispanic White and non-Hispanic Black individuals is that weathering does not account for environmental exposures or other stressors across generations, which may be important in understanding cumulative risks. Extending the weathering hypothesis by looking beyond an individual's life course to include intergenerational exposures may be important in understanding risks in subsequent generations among non-Hispanic Black families.

**Figure 1:**
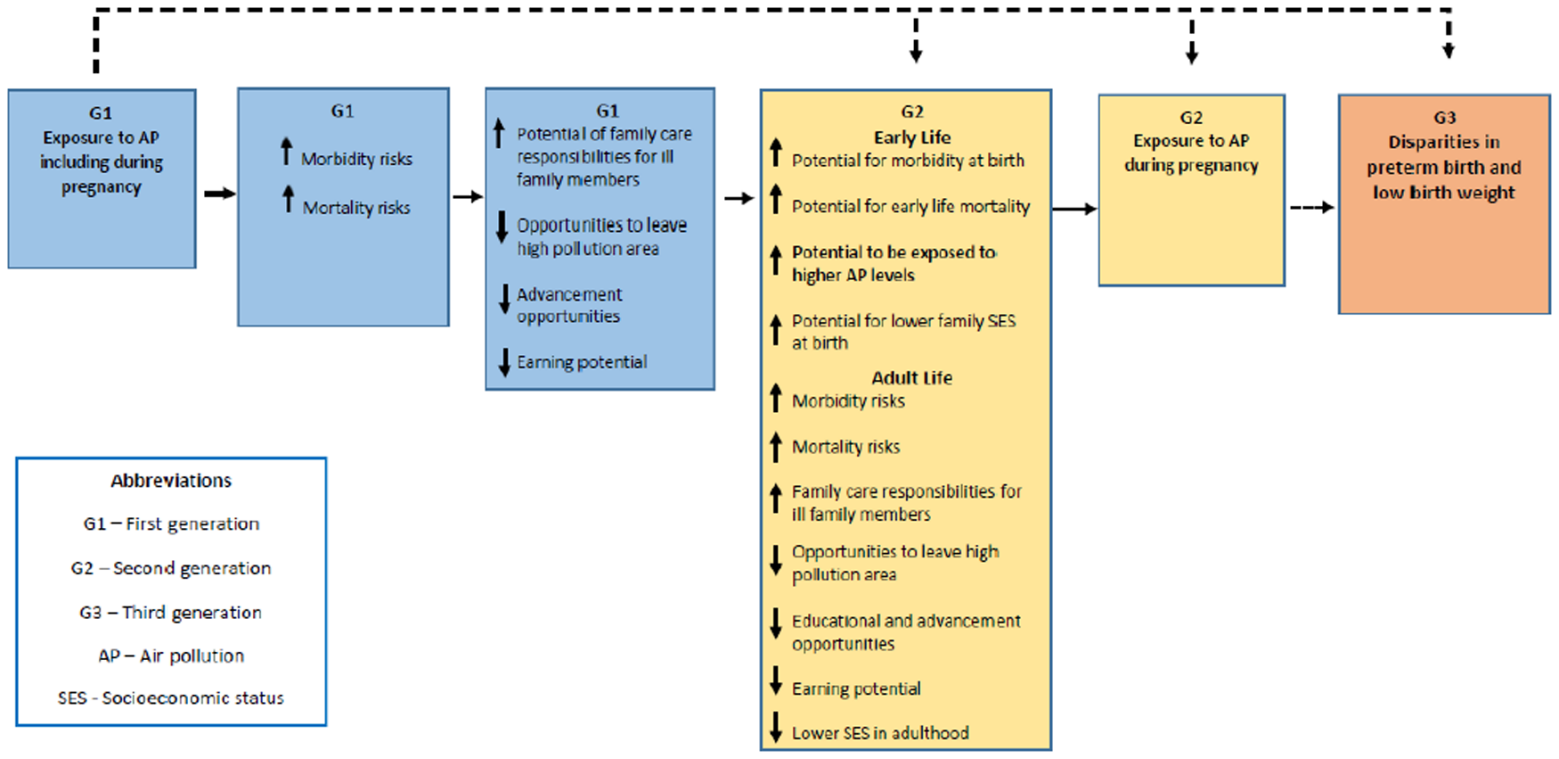
Multi-generational exposure to air pollution and adverse birth outcomes—conceptual framework.

Potential links between air pollution exposure and adverse birth outcomes may lead to the perpetuation of increased risks among subsequent generations and contribute to sustained disparities across generations. For example, low birth weight and preterm birth rates are higher among individuals who had lower than normal weight at birth or who were born prior to 37 weeks of gestation (36), and intergenerational associations have been reported (37, 38). Numerous studies conducted to date have evaluated long-term or pregnancy-wide exposure to air pollution and birth outcomes within a single generation (39). Others have evaluated pre- and postnatal exposures on child health outcomes (40). However, exposure to air pollution may have health impacts across generations that may not be fully accounted for using only prenatal exposures or exposures within a given generation. Examining intergenerational air pollution exposure may be helpful for understanding racial and ethnic health disparities in subsequent generations and help account for yet unexplained racial and ethnic differences in adverse birth outcomes. Associations may operate via multiple pathways that include increased potential for morbidity during the life course, and reduced socioeconomic advancement opportunities among families. For example, Sharkey found that individuals whose childhood residence was in the lowest quartile of income, which are disproportionately Black individuals, tended to live in similar neighborhoods as adults, suggesting that neighborhood disadvantage can be “inherited” across generations (41). Neighborhood income and racial composition, as well, are associated with higher levels of air pollution as well as potentially toxic components of pollution (42), and these disparities can persist over time and contribute to illness. Morbidity during the life course may subsequently contribute to lower socio-economic status (SES) of the mother and other caregivers at the birth of the next generation, and increased risks of adverse birth and child health outcomes in subsequent generations (Figure 1). In addition, multiple pathways may involve direct and indirect effects, including epigenetic changes in germline cells that have been reported for air pollution and other environmental exposures (43, 44), that work together to produce adverse health outcomes in subsequent generations. Germline cells lead to the development of the reproductive cells in females and males and may lead to the transfer of altered gene expression across generations.

## Examples of data sources in the U.S. for examining intergenerational associations

### Health outcomes

Researchers interested in conducting studies to examine intergenerational associations can benefit from using existing data resources, such as linked birth registries data or existing cohorts. In other instances, opportunities may exist to work with state level or federal agencies to obtain restricted identifiable data. To date, a number of studies have reported analyses using intergenerational datasets; these datasets may provide opportunities to investigate the role of structural racism on environmental exposures and adverse health outcomes.

One example of such a dataset that provides potential opportunities to investigate intergenerational associations is the South Carolina birth outcome data and multigenerational dataset. This is an integrative dataset that includes data obtained from singleton birth certificates for the state of South Carolina from 1989 to 2014 (45). Birth data are geocoded at the census tract level and linked along the maternal line and span three generations. In the U.S., census tracts are statistical subdivisions of varying geographical area that include an average population size of 4,000 inhabitants, range 1,200–8,000 inhabitants (U.S Census Bureau). The resulting structure of the data includes grandmothers (G1), mothers (G2), and G2's children (G3) within the same family. Birth outcomes are available in this dataset as this information is routinely collected at birth and recorded on birth certificates. A description of this multigenerational dataset for 1989–2014 has been previously reported (45) and the dataset includes 60,258 linked families (family trees), with 32,046 for non-Hispanic Black infants and 28,212 for non-Hispanic White infants.

In addition to the South Carolina birth outcome data and multigenerational dataset, other states have produced similar multigenerational datasets. Two of those intergenerational datasets cover periods in the early 1980s for which census tract level air pollution estimates are available for at least a part of the older generation life span. First, using data from the State of Washington, a database was created of vital statistics data that were subsequently linked to birth certificate data of mothers born in Washington State (46). The resulting dataset included 46,000 births that occurred between 1987 and 1995 to mothers who were born between 1949 and 79 and who were of the following racial and ethnic groups: African American, Hispanic, Native American and White individuals. Second, an intergenerational dataset was generated by linking the State of Virginia live birth data from 2005 to 2009 to mothers born during 1960–1997. This overall dataset included 170,624 records, but a subset that was restricted to non-Hispanic Black and non-Hispanic White first-born singleton infants included 69,702 records (47).

In addition, a number of multigenerational studies (48, 49), have been established and may serve as either potential data sources or methodological inspiration for future studies. Furthermore, future studies exploring the logistical complexity of assembling a cohort of three generations could incorporate the design of the Nurses' Health Study II (50). In the Nurses' Health Study II, both historical and current data were collected, thereby enabling the utilization of data on three generations. Specifically, enrolled participants represent the second generation or G2 and information was gathered on G1 and G3. These datasets point to existing opportunities and highlight methodology that may be applied to different states with other characteristics of interest.

The intergenerational data format described here offers an advantage over single point-in-time measures of structural racism or environmental exposures and may elucidate pathways leading to adverse health outcomes. Using intergenerational information may inform whether maternal exposure to structural racism impacts health outcomes through shared exposure (i.e., inherited disadvantage) or through biological pathways (i.e., a mother's birthweight is a determinant of her offspring's birthweight). An important consideration is the statistical approach that would be appropriate for intergenerational studies and effectively account for both historical and ongoing exposures. Mediation analyses have been previously used to understand direct and indirect effects in studies examining smoking transmission across three generations (51, 52), and such an approach would be useful for studies that might be conducted with the data described here. For example, in one study that examined smoking transmission across three generations over a 45-year period, the study team utilized a multi-step approach (52). First, logistic regression models were utilized to evaluate direct relationships for each of the following pairs: associations between the first generation (G1) and second generation (G2) smoking, G2 and the third generation (G3) smoking, and G1 and G3 smoking. Second, the investigators examined whether transmission of smoking between the grandparents (G1) and grandchildren (G3) was mediated by G2 smoking using a Sobel test that incorporated standardized coefficients resulting from the logistic regression models. Another approach could be the use of multilevel logistic regression to account for clustering resulting from the use of census tract air pollution data. Pollutant may be represented by typologies that could be used to create a composite measure of air pollution for G1 and G2. Based on the distribution of the data, a cutoff value could be used to create high and low categories for G1 and G2, separately. Combining the resulting data would form the following categories G1 high/G2 high, G1 high/G2 low, G1 low/G2 high, and G1 low/G2 low (45). In addition, the large dataset afforded by using available birth data over a long period would allow for examination of the joint effects of G1 and G2 prenatal exposure to air pollution on adverse birth outcomes among G3.

### Air pollution

The United States Environmental Protection Agency (U.S EPA) routinely collects data on air pollution. The U.S EPA compiles data from states reporting hourly air pollution concentrations at approximately 5,000 monitors across the country (53) and these historical data (currently available from 1980 to 2022) are publicly available to be linked to existing or even newly created intergenerational datasets. Additionally, other groups have compiled EPA and other data using modeling. For example, the Center for Air, Climate, and Energy Solutions (CACES) is a research center staffed by researchers from multiple disciplines and institutions.

**Figure 2:**
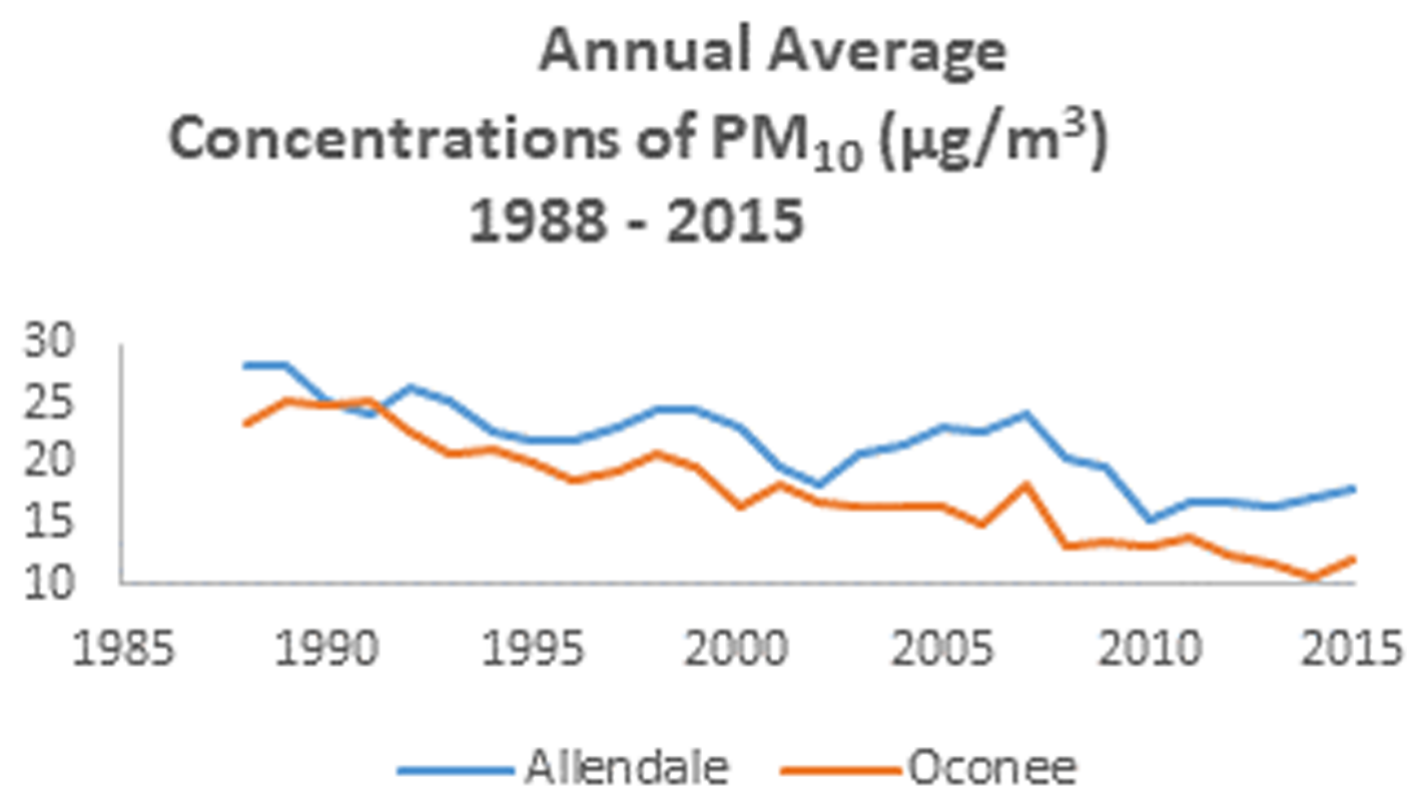
Annual average concentrations of PM_10_(μg/m^3^) 1988–2015.

The CACES website has air pollution data that can be easily requested in a few steps, the process does not require a lengthy processing time, and the data timeframe supports examination of intergenerational exposures. Data availability from CACES varies across pollutants, but the earliest year is 1979, which would make this data source compatible with studies interested in at least two generations. This data source is particularly useful for studies interested in average exposure as the data are available as annual estimates. CACES focuses on key themes about air pollution, including regional differences of air pollution. Additionally, CACES incorporates measurement and modeling approaches that are helpful for investigating both spatial and temporal differences in exposure and associated outcomes. Information on CACES and their air pollution data may be accessed at the following website (www.caces.us/data). Data available at CACES can be used to estimate annual air pollution exposure for individual participants at the census tract level. CACES produces air pollution estimates for criteria air pollutants, the air pollutants for which the U.S. EPA has established standards to protect human health. Criteria pollutants include carbon monoxide (CO), ozone (O_3_), particulate matter ≤10, ≤2.5 μm in aerodynamic diameter (PM_10_, PM_2.5_), nitrogen dioxide (NO_2_), and sulfur dioxide (SO_2_). According to CACES, average annual estimates, except for ozone, were derived using models that incorporate data from U.S. EPA regulatory monitors, satellite-derived air pollution estimates of air pollution to account for locations with missing data, and information on land use. For ozone, estimates are based on the daily maximum 8-hour moving average concentrations for the period May through September (54), which corresponds to the warmer months of the year (55). Figure 2 shows a sample of air pollution data obtained from CACES using PM_10_ from 1989 to 2015 for two census tracts in South Carolina. The census tracts shown are in Allendale [Federal Information Processing Standards (FIPS) code: 45005970200] and Oconee (FIPS code: 45073030602). These census tracts were selected to compare a census tract with a high percent of non-Hispanic White population to a high percent non-Hispanic Black population. According to 2010 census data available on Social Explorer (https://www.socialexplorer.com/explore-tables), which provides access to current and historical U.S census data, the population for the census tract in Allendale was 78.4% Black and the population for the census tract in Oconee was 88.4% White. These data can be generated for any census tract in the United States and annual concentrations of air pollutants can be compared over time. Data are available for O_3_, SO_2_, NO_2_, for 1979–2017, PM_10_ for 1988–2017, CO 1990–2017 and PM_2.5_ from 1999 to 2017.

Another source of historical air pollution data is from the Atmospheric Composition Analysis Group at Washington University in St. Louis. This source has monthly and annual measures for fine particulate matter which are available beginning from 1981 (56). This data source is particularly useful for studies examining time varying exposures and outcomes that might be impacted by windows of susceptibility that vary over time because it includes data at a finer temporal scale (monthly estimates) compared to the CACES data (annual estimates). The spatial resolution is 1 km by 1 km grid squares. Other sources of historical air pollution data that allow for evaluating time varying exposures and windows of susceptibility over the gestational period are also publicly available. For example, using data from the U.S EPA Air Quality System (AQS) and other publicly available data sources including meteorological and land use data, daily air pollution data for different periods of gestation can be estimated at the census tract level based on methods used in long term studies of air pollution (57). Data from the EPA AQS can be used to estimate daily prenatal exposure (versus the average annual concentrations in the CACES data). This provides a distinct advantage in that it accounts for variations in pollutant levels at different points in the year as well as potential windows of susceptibility during pregnancy, such as trimester specific exposure, or exposures from shorter times frames, such as weeks or days before delivery (58). Methods to assign exposure estimates of census tract-level concentrations are described in Di et al. (57). In addition to recommendations to use multilevel data and incorporate the life course and time, and, as we have discussed here, across generations, a major advantage of using such datasets, especially birth registry data, is that they are sufficiently large and adequately powered to allow for the evaluation of effect modification and subgroup analyses.

Although the historical air pollution data sources listed above are, in some cases, readily available, they are not without limitation. First, attempting to document air pollution exposure over long periods of time is a challenge. Challenges include availability of data to cover the entire timeframe of interest or if using data from multiple sources, potential differences in measurement methodology might be an issue. Second, an important limitation is that air pollution estimates using only data from the U.S EPA may be based on monitors that are placed in a central location and the same estimates may be assigned to individuals who live near a sited monitor and to individuals who live miles away from a monitor if using census tract level estimates. Modelling, which takes into account other information such as land use and satellite data (54), is used in some cases and may improve estimates. However, historical data do not take into account daily activity patterns (i.e., time spent outside vs. inside) as has been done in prospective studies (59) or adjust for time that individuals may be at locations other than at home (60). In other cases, kriging, an interpolation method that takes into account available data, including address information and distance to available monitors, to improve air pollution estimates, may be used (61). Third, these limitations may influence study results particularly if the misclassification varies by groups and caution should be taken when interpreting and discussing results. Nevertheless, utilizing the above data sources will provide unique opportunities to examine factors that may contribute to health disparities. Equally important, studies using data sources listed above may be important in identifying gaps for future prospective studies where air pollution exposure assessments may be improved.

### Summary

Combining long-term air pollution and other environmental data and linked multi-generational health outcome data is essential for environmental epidemiologists interested in estimating associations between air pollution and intergenerational social, health and economic disparities. Further, evaluating pathways by which environmental and structural racism could play a role in these associations, and incorporating available metrics that reflect the historical underpinnings of the systems in our society that result in unfair exposures and health burdens, has been called for on multiple fronts. Insights obtained from this kind of study may be useful for understanding persistently observed disparities in adverse birth outcomes, and intervening to eliminate them.

## Discussion

A fundamental component to advancing health equity is a need to design rigorous research studies that quantify structural racism such that it accurately captures the multi-dimensional way it affects the lives of minoritized individuals. The data sources presented here provide opportunities to examine associations between exposure to air pollution, which may be driven by structurally racist policies and practices, and adverse birth outcomes. These opportunities can be expanded to include maternal outcomes as well as other outcomes that have a persistent disparity between minoritized and non-minoritized groups. In addition, these steps highlight a need to focus less on individual-level factors associated with adverse outcomes and more on systematic attempts at correcting the systems that enable structurally racist practices to flourish.

Consistent with recent calls to enhance the rigor and care with which hypotheses around race as an effect modifier in environmental epidemiology are developed and findings explained (22, 23), we suggest that researchers can tap into existing data and frame their research in a way that points toward policy and interventions to reduce health disparities. This suggestion is consistent with a recent exhortation to move beyond characterizing race/ethnic disparities in air pollution to addressing them (62). Others have called for combining antiracist principles with those of community-based participatory research, an approach that emphasizes community knowledge and equal partnerships with academics (63). Collaborating in research with affected communities and co-analysis of collected data will lead to enhanced insights into causal mechanisms being explored (64). Thus, in addition to tapping into existing data sources and applying methods such as those we illustrate here, we advocate for engaging community members and practitioners in evaluating and framing research questions. Such approaches may be useful for providing a comprehensive understanding of the cumulative nature of environmental exposures and provide actionable information on how public health policy and action can be tailored.
